# Procalcitonin as a biomarker for postoperative pneumonia: a study on dynamics following cardiopulmonary bypass in adults

**DOI:** 10.1186/s12872-025-04654-3

**Published:** 2025-04-30

**Authors:** Guang-Xu Mao, Li-Yun Wang, Wen-Sen Chen, Sheng Zhao, Yong-Feng Shao, Yu-Zhen Guan, Zhen Lu, Feng Zang

**Affiliations:** 1 Department of Infection Management, The First Affiliated Hospital with Nanjing Medical University, Nanjing, Jiangsu 210029 China; 2 Department of Infection Management, Xinghua People’s Hospital Affiliated to Yangzhou University, Xinghua, Jiangsu 225700 China; 3 Department of Cardiovascular Surgery, The First Affiliated Hospital with Nanjing Medical University, Nanjing, Jiangsu 210029 China

**Keywords:** Cardiopulmonary bypass, Cardiac surgery, Postoperative pneumonia, Procalcitonin

## Abstract

**Objective:**

Postoperative pneumonia (POP) frequently complicates cardiac surgery that involves cardiopulmonary bypass (CPB). This study aimed to assess the diagnostic utility of procalcitonin (PCT) for identifying pneumonia after CPB-assisted cardiac surgery.

**Methods:**

Patients diagnosed with POP were enrolled in the retrospective matched case-control study and were admitted to a Grade III general hospital in Nanjing in 2023. POP diagnosis was determined based on a combination of clinical and microbiological criteria.PCT and white blood cell count (WBC) data were systematically collected from day 1 (T1) to day 5 (T5). Receiver operating characteristic (ROC) curve analysis and subject operating characteristics were utilized to evaluate the diagnostic performance of biomarkers. At the same time, a binary logistic regression model was developed to identify factors that influence the diagnosis of POP.

**Results:**

The study included 220 age- and sex-matched patients, comprising 56 individuals with POP and 164 uninfected patients constituting the non-POP group. ROC curve analysis revealed that serum PCT concentration exhibited an AUC > 0.7 from day 2 to day 5, whereas other indices demonstrated AUCs < 0.7 at these time points. Univariate and multivariate analyses highlighted serum PCT concentration on day 2, WBC count on day 5, the PCTT4-T1 variation rate, and days of mechanical ventilation as significant predictive factors for POP diagnosis, each demonstrating statistical significance (*P* < 0.05). The calculated AUC was 0.837 (95%CI: 0.773–0.902). The absolute PCT value exhibited superior diagnostic performance relative to its variance rate and WBC count, yielding optimal diagnostic accuracy with a cutoff value of 3.45 ng/ml.

**Conclusion:**

Serum PCT absolute value demonstrates higher sensitivity and specificity than other indices, offering superior diagnostic potential for predicting POP.

**Supplementary Information:**

The online version contains supplementary material available at 10.1186/s12872-025-04654-3.

## Perspective

Procalcitonin (PCT) levels showed promise as a sensitive and specific marker for predicting postoperative pneumonia (POP) following cardiac surgery with cardiopulmonary bypass (CPB).

## Introduction

Cardiopulmonary bypass (CPB) is recognized as a foundational technique in contemporary cardiac surgery, offering significant advancements in patient prognosis and broadening surgical possibilities [1]. However, the application of CPB was associated with inherent risks, triggering a cascade of physiological and pathological responses that include systemic inflammation, immune system disruption, and changes in multi-organ function [2]. These responses can lead to a spectrum of postoperative complications, notably pulmonary infections, which significantly impact the patient’s recovery and long-term prognosis. Given the severity and prevalence of early pneumonia, an accurate diagnosis is crucial for prompt therapeutic interventions and improving patient prognosis. Regrettably, diagnosing postoperative pneumonia (POP) remains challenging in current clinical practice, mainly due to the lack of specific and sensitive biomarkers [3]. Consequently, identifying novel biomarkers to diagnose early POP accurately has become an urgent research priority within the medical community. In this context, procalcitonin (PCT) has emerged as a promising biomarker, attracting increasing interest among researchers. Serum PCT concentrations are exceedingly low in healthy individuals. However, they significantly increase during bacterial infections, making PCT a robust candidate for diagnosing such conditions [4]. Previous studies have shown the superior specificity of PCT over traditional inflammatory markers in indicating postoperative infections; however, the patterns of PCT changes following adult extracorporeal cardiac surgery and its potential for early pneumonia diagnosis have not been thoroughly studied. The existing literature offers divergent interpretations regarding changes in PCT levels, and a comprehensive validation of its sensitivity and specificity in diagnosing early pneumonia is still lacking [5, 6]. Therefore, this study aims to assess the natural progression of PCT changes after extracorporeal cardiac surgery in adults and to explore its potential in diagnosing early pneumonia. We anticipate that this investigation will yield a more precise diagnostic tool for clinical use while laying a solid scientific groundwork for infection management and therapeutic decision-making after cardiac surgery, thus informing early intervention strategies and ultimately improving patient prognosis.

## Materials and methods

### Study design and sample

This study utilized a matched case-control design. Following approval from the Medical Ethics Committee of the First Affiliated Hospital of Nanjing Medical University (2024-SR-535), this study included patients admitted to the Department of Cardiac and Major Vascular Surgery in 2023. These patients developed POP within seven days following CPB cardiac surgery and were matched with a control group in a 1:3 ratio based on age and gender. Inclusion criteria were: (1) adult patients aged 18 to 80 years undergoing CPB cardiac surgery and (2) availability of comprehensive laboratory test data. Exclusion criteria included: (1) preoperative temperature ≥ 38 °C; (2) cardiac surgery performed due to trauma, infective endocarditis, tumor, malignancy, or emergency; and (3) diagnosis of any other infectious disease (e.g., surgical site infection, sepsis), 220 patients met the inclusion criteria: 56 in the POP group and 164 in the non-POP group, matched by age and sex, without hospital-acquired infections. We utilized PASS (15.0) to calculate the required sample size. Based on the ROC curve and AUC analysis, we determined that a minimum sample size of 172 patients was necessary, which included 43 cases of postoperative pneumonitis. Our study successfully met this minimum sample size requirement.

### Data collection

1.2.1 Patient baseline data were collected via the hospital’s electronic case system, including demographic characteristics, preoperative diagnoses, surgical methods, duration of mechanical ventilation, ICU stays, length of hospital stay, and microbiological examination results. The intraoperative CPB manual registry obtained Times for CPB and aortic cross-clamp. Serum PCT concentrations and WBC counts were meticulously recorded from postoperative day 1 to day 5 (T1 to T5) for each patient. The PCT variability rate was calculated using the formula [7]: (PCT_*delayed*_−PCT_*T1*_)/PCT_*T1*_ × 100%. PCT_*delayed*_ represents the serum PCT concentration measured from T2 to T5, with negative values signifying a decrease in PCT concentration. All data were collected by the Xinglin Real-Time Nosocomial Infection System and Intelligent Integrated Health (IIH) platform.

1.2.2 Preoperative nutritional evaluation: BMI and CONUT indicators were assessed based on the data. (1) BMI [8]: BMI (kg/m²) = weight (kg) / height² (m); a BMI of less than 18.5 kg/m^2^ is classified as excessively light, a BMI between 18.5 kg/m^2^ and 24 kg/m^2^ is categorized as usual, a BMI between 24 kg/m^2^ and 28 kg/m^2^ is deemed overweight, and a BMI of 28 kg/m^2^ or greater is classified as obese. (2) CONUT Score [9, 10]: Calculated based on serum albumin (ALB), total cholesterol (TC), and lymphocyte count (TLC). Scores are assigned: For ALB, ≥ 35 g/L scores 0, 30–34.9 g/L scores 2, 25–29.9 g/L scores 4, < 25 g/L scores 6. For TC, ≥ 4.68mmol/L scores 0, 3.64-4.67mmol/L scores 1, 2.60-3.63mmol/L scores 2, < 2.60mmol/L scores 3. For TLC, ≥ 1.60 × 10⁹/L scores 0, 1.20–1.59 × 10⁹/L scores 1, 0.80–1.19 × 10⁹/L scores 2, < 0.80 × 10⁹/L scores 3. The total score determines nutritional status: 0–1 indicates normal nutrition, 2–4 indicates mild malnutrition, 5–8 indicates moderate malnutrition, and 9–12 indicates severe malnutrition.

### Diagnostic criteria for pneumonia

The primary outcome of this study concerned the incidence of pneumonia within 7 days post-surgery. Diagnosis of POP was based on the 2018 CDC diagnostic criteria for pneumonia [11, 12], outlined as follows: (1) Presence of at least one of the following on a minimum of two chest radiographs (only one chest radiograph is necessary for patients lacking underlying cardiopulmonary disease): (a) New or progressive persistent infiltrative shadows; (b) Solid lesions; (c) Cavitation; (2) Fulfillment of at least one of the following criteria: (a) Unexplained fever > 38 °C; (b) WBC count decreased (< 4 × 10^9/L) or increased (> 12 × 10^9/L); (c) Unexplained change in mental status in adults aged 70 years or older; (3) At least two of the following criteria are met: (a) New onset of purulent sputum, change in sputum character, increase in respiratory secretions, or increased need for suctioning; (b) New or worsening cough, dyspnoea, or shortness of breath; (c) Lung rales or bronchial breath sounds; (d) Deterioration in gas exchange function (hypoxemia, increased oxygen demand, or increased ventilator requirements).In addition, the results of pathogenic cultures serve as a foundational basis for diagnosing POP.

### Perioperative management

All surgeries were conducted via median sternotomy. CPB was utilized in all patients. The ascending aorta was cannulated with an appropriately sized cannula. Venous cannulation was selected with separate cannulas in the superior and inferior vena cava. Cardiac arrest fluids primarily consisted of Histidine-mono-tryptophan-mono-ketoglutarate arrest fluid (HTK) and Del Nido cardiac arrest fluid (DN), which have been demonstrated in multiple studies to offer the most cardioprotective and ease of perfusion, thereby optimizing the outcome and simplifying the surgical procedure [13]. Perioperative prophylactic antibiotic therapy is routinely administered to prevent infection, with the first dose of antibiotics being administered intravenously within 60 min of incision and subsequently every 3 to 4 h during surgery. Additionally, we continue administering adequate antibiotics according to the guidelines for 48 h postoperatively. Cefuroxime is typically selected as a prophylactic IV antibiotic. According to the administration guidelines for cefuroxime, the recommended dosage for adults is 1.5 g per administration.

### Statistical analyses

Using IBM SPSS 29 statistical software, continuous variables were assessed for normal distribution via the Shapiro-Wilk test; variables adhering to a normal distribution were presented as mean ± standard deviation (SD), while comparisons between groups of continuous variables utilized the t-test. Measures indicative of skewed distributions were depicted as median (interquartile range [IQR]), and comparisons were conducted using the Mann-Whitney U test. Count data were represented as n (%), with inter-group comparisons conducted via the χ2 test. ROC curves and AUC were employed to evaluate the POP diagnostic utility of WBC, PCT, and PCT variants. Logistic multivariate regression models were utilized to assess postoperative factors independently predictive of POP. All reported P-values were two-sided, with *P* < 0.05 considered indicative of statistical significance.

## Results

### Incidence of pneumonia after cardiac surgery

Over the past three years, the prevalence of cardiac macrovascular POP has been 5.87%, 5.90%, and 3.99%, respectively. The proportions of POP within hospital-acquired infections have been 78.39%, 81.82%, and 75%, respectively. Notably, a significant decrease in the incidence of POP was observed in 2023 (*P* < 0.05), whereas no statistically significant difference was noted in the component ratios (*P* > 0.05) (Table 1).

**Table 1:** Incidence of pneumonia after cardiac surgery

Times	Rate of infection			Component ratio	
	Number of discharges	Infection rate *n* (%)		General infection	Component ratio (%)
2021	2162	127 (5.87)		162	127(78.39)
2022	2287	135 (5.90)		165	135(81.82)
2023	2632	105 (3.99)		140	105(75.00)
*χ* ^*2*^	12.145			2.098	
P-value	0.002			0.350	

### Baseline patient characteristics

No significant differences were observed between the two groups of patients regarding age, gender, BMI, underlying diseases, types of cardiac surgery, smoking status, alcohol consumption, and preoperative nutritional scores (CONUT). A longer duration of CPB and a longer duration of aortic cross-clamp were significantly associated with the development of POP in the POP group (*P* < 0.05). Patients in the POP group underwent mechanical ventilation, required intensive care, and experienced hospitalization for significantly more extended periods (*P* < 0.001) (Table 2).

**Table 2:** Comparison of clinical baseline data between the two groups of patients

variant		POP Group (*n* = 56)	Uninfection group (*n* = 164)	χ^2^ /Z value	*P*-value
Sex: Male		39 (69.64)	102 (62.19)	1.006	0.316
Age (years)					
	< 30	0(0)	1 (0.61)	3.393	0.335
	30 ~ 49	6 (10.71)	16 (9.76)		
	50 ~ 69	31 (55.36)	110 (67.07)		
	>=70	19 (33.93)	37 (22.56)		
BMI					
	too light	2 (3.57)	7 (4.27)	1.288	0.732
	normalcy	29 (51.78)	71 (43.29)		
	overweight	17 (30.36)	61 (37.19)		
	obese	8 (14.29)	25 (15.24)		
Smoking history: Yes		22 (39.28)	50 (30.49)	1.468	0.226
Drinking history: Yes		12 (21.43)	49 (29.88)	1.487	0.223
Complication (medicine)					
	History of hypertension: Yes	23 (41.07)	56 (34.15)	0.870	0.351
	History of diabetes: Yes	8 (14.28)	13 (7.93)	1.955	0.162
	Cerebrovascular diseases: Yes	8 (14.28)	18 (10.97)	0.439	0.508
CONUT score					
	Normal nutrition	18 (32.14)	68 (41.46)	6.214	0.102
	mild malnutrition	32 (57.14)	90 (54.88)		
	Moderate malnutrition	5 (8.93)	6 (3.66)		
	severe malnutrition	1 (1.79)	0(0)		
Types of Cardiovascular Surgery					
	coronary artery	9 (16.07)	17 (10.36)	2.094	0.718
	valve surgery	35 (62.50)	114 (69.51)		
	Aortic surgery	5 (8.93)	13 (7.93)		
	congenital heart disease	2 (3.57)	9 (5.49)		
	the rest	5 (8.93)	11 (6.71)		
CPB time (min)		142 (117, 179)	128 (106, 160)	-2.406	0.016
Aortic cross-clamp time(min)		100 (82, 128)	86 (67, 112)	-2.919	0.004
Mechanical ventilation (days)		5(2,12.75)	2(1,3)	-5.652	< 0.001
ICU (days)		10(6,19)	6(5,8)	-4.192	< 0.001
Postoperative antimicrobial use (days)		18(13,23)	12(9,15)	-5.565	< 0.001
Length of stay (days)		27(20,36)	20(16,25)	-4.370	< 0.001

### Postoperative kinetics of WBC, PCT, and PCT variability in the two groups of patients

**Fig. 1:**
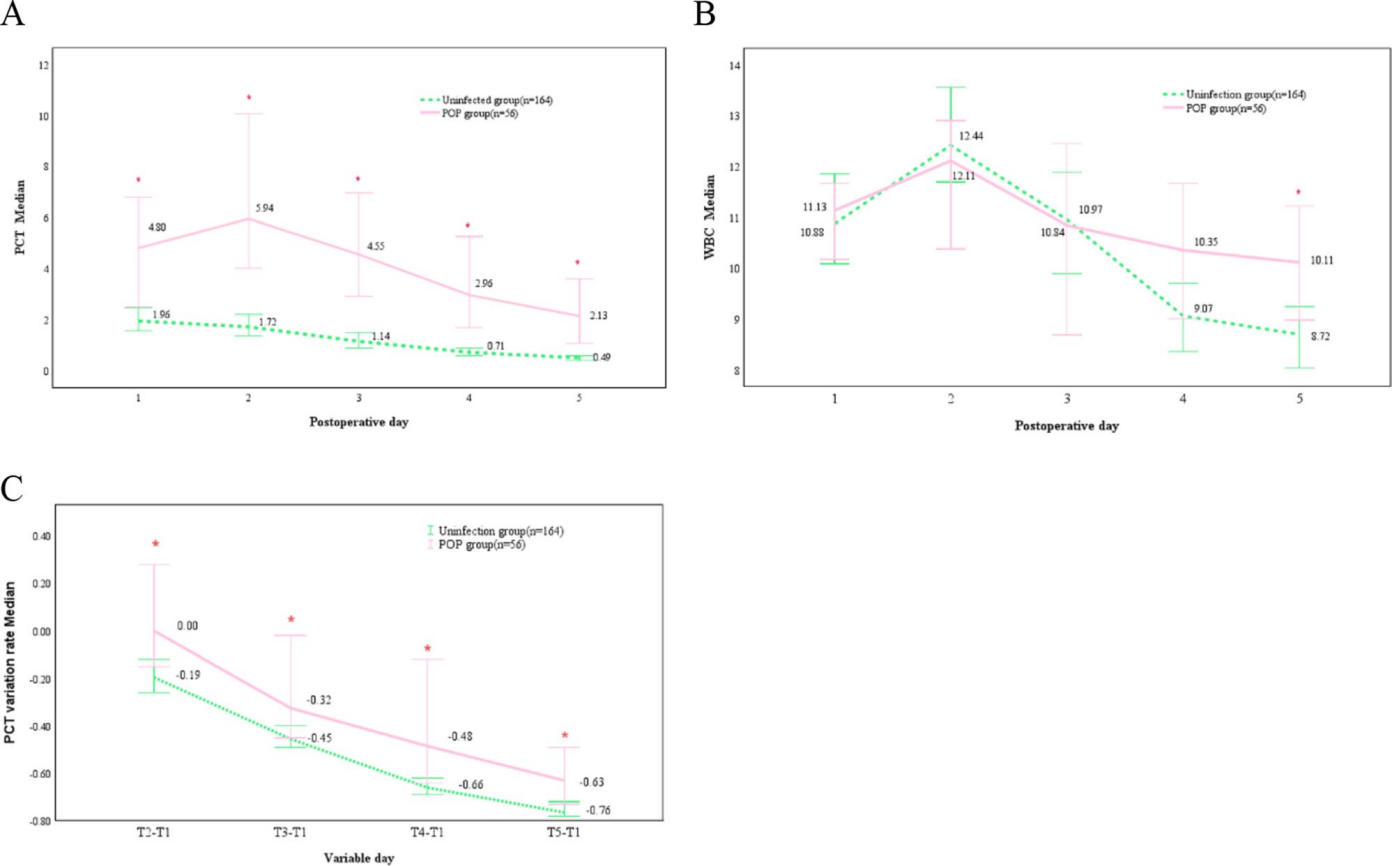
Kinetic changes in PCT (**A**), WBC (**B**), and PCT variability (**C**) in the POP group (solid line) and uninfected patients (dashed line) over the five postoperative days, with asterisks denoting statistical differences between groups (*P* < 0.05)

**Fig. 2:**
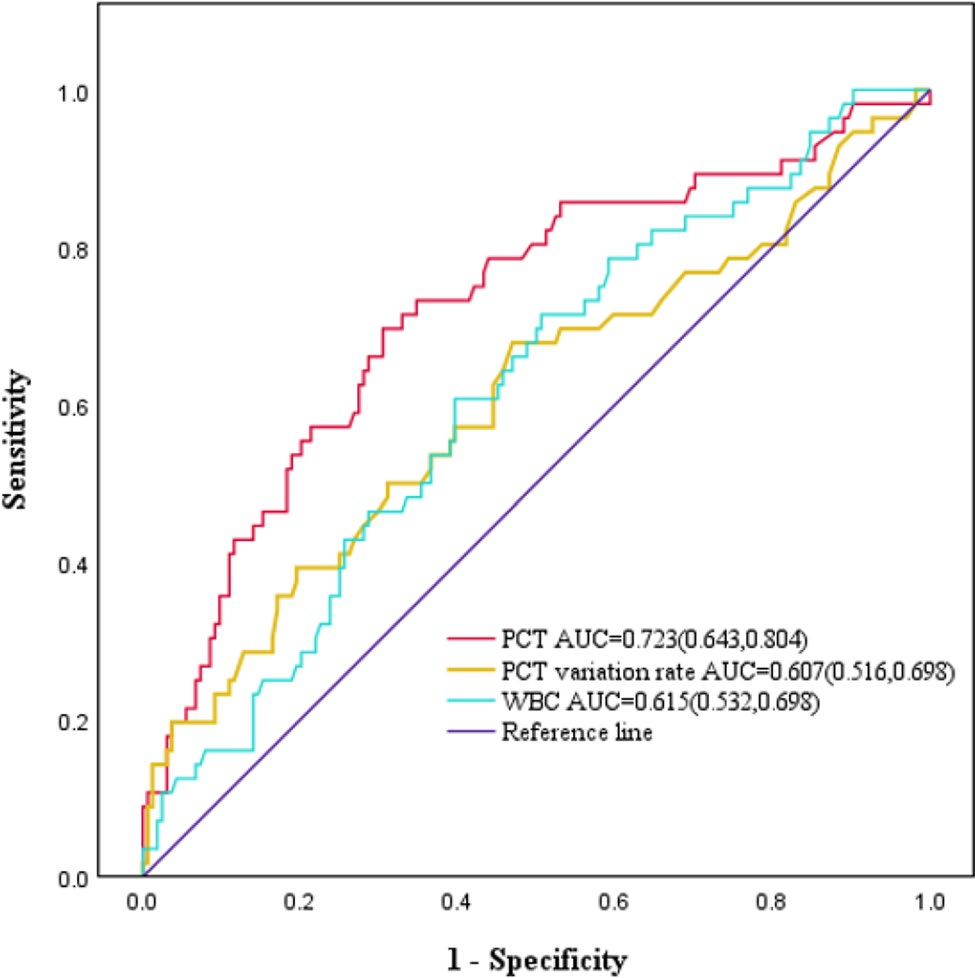
Comparison of ROC curves showing the predictive value of PCT (value on T2), PCT variation (value on T2), and CRP (value on T5) for the diagnosis of POP

Changes in the indexes were observed postoperatively from T1 to T5. Serum PCT concentrations increased and decreased in the infection group at each postoperative time point, reaching the peak on the 2nd postoperative day. In contrast, the control group exhibited a gradual decrease. Furthermore, the concentration of PCT in the infection group was consistently and significantly higher than that in the control group at all time points, with statistically significant differences (*P* < 0.05). The WBC count peaked on the 2nd postoperative day. Notably, an important difference was only observed in patients infected on the 5th postoperative day (*P* < 0.05). The PCT variability rate was notably higher in the infected groups compared to the control group (*P* < 0.05) (Table 3; Fig. 1). ROC curves indicated that serum PCT concentrations had an AUC > 0.7 from T2 to T5, and an AUC < 0.7 for the remaining points (Table 4). Results indicated that the diagnostic utility of serum PCT concentrations was superior to both the PCT variability rate and WBC count (Fig. 2).

**Table 3:** Comparative analysis of postoperative PCT, leukocyte, and PCT clearance changes and indicators in the two groups of patients

variant	POP Group *n* = 56	Uninfection group *n* = 164	Z-value	*P*-value
PCT (ng/mL)				
T_1_	4.80 (1.39, 4.80)	1.96 (0.85,5.14)	-3.188	0.001
T_2_	5.94 (2.06,13.15)	1.72 (0.76,4.42)	-4.987	< 0.001
T_3_	4.55 (1.35,10.78)	1.14 (0.52,2.90)	-5.174	< 0.001
T_4_	2.96 (1.05,8.25)	0.71 (0.33,1.95)	-5.370	< 0.001
T_5_	2.13 (0.73,6.43)	0.49 (0.21,1.18)	-6.005	< 0.001
WBC (10^9/L)				
T_1_	11.13 (8.89, 13.21)	10.88 (8.94, 13.58)	-0.091	0.927
T_2_	12.11 (8.76,14.53)	12.44 (10.12, 16.67)	-1.677	0.094
T_3_	10.84 (7.74,13.32)	10.97 (8.17, 13.30)	-0.354	0.723
T_4_	10.35 (7.74,13.06)	9.07 (7.39, 12.10)	-1.698	0.089
T_5_	10.11 (7.97, 12.55)	8.72 (6.64, 11.27)	-2.571	0.010
PCT variation rate				
T_2_ -T_1_	0.00(-0.31,0.58)	-0.19 (-0.35,0.14)	-2.384	0.017
T_3_ -T_1_	-0.32(-0.53,0.69)	-0.45(-0.62,0.18)	-2.699	0.007
T_4_ -T_1_	-0.48 (-0.70,0.71)	-0.66 (-0.77,0.39)	-3.012	0.003
T_5_ -T_1_	-0.63(-0.78,0.06)	-0.76(-0.86,0.60)	-3.656	< 0.001

**Table 4:** Comparison of AUC and efficiency of PCT, WBC, and PCT clearance in diagnosing postoperative infections

variant	AUC (95% CI)	Cut-off	Sensitivity	Specificity	Jordon index
PCT (ng/mL)					
T_1_	0.643 (0.555,0.730)	3.845	0.589	0.707	0.297
T_2_	0.723 (0.643,0.804)	3.450	0.696	0.695	0.392
T_3_	0.732 (0.650,0.813)	2.650	0.661	0.744	0.405
T_4_	0.740 (0.661,0.820)	1.325	0.732	0.665	0.397
T_5_	0.769 (0.693,0.844)	0.700	0.786	0.640	0.426
WBC (10^9/L)					
T_1_	0.496 (0.408,0.584)	9.880	0.679	0.396	0.075
T_2_	0.425 (0.337,0.513)	25.820	0.018	0.982	0.000
T_3_	0.484(0.394,0.574)	12.445	0.375	0.677	0.052
T_4_	0.576 (0.490,0.662)	10.130	0.554	0.628	0.182
T_5_	0.615(0.532,0.698)	9.435	0.607	0.604	0.211
PCT variation rate					
T_2_ -T_1_	0.607 (0.516,0.698)	20.970	0.018	0.994	0.012
T_3_ -T_1_	0.621(0.532,0.710)	-0.155	0.429	0.774	0.203
T_4_ -T_1_	0.635(0.544,0.725)	-0.265	0.429	0.829	0.258
T_5_ -T_1_	0.664 (0.579,0.748)	-0.695	0.607	0.646	0.253

### Analysis of risk factors for pneumonia after CPB cardiac surgery

**Fig. 3:**
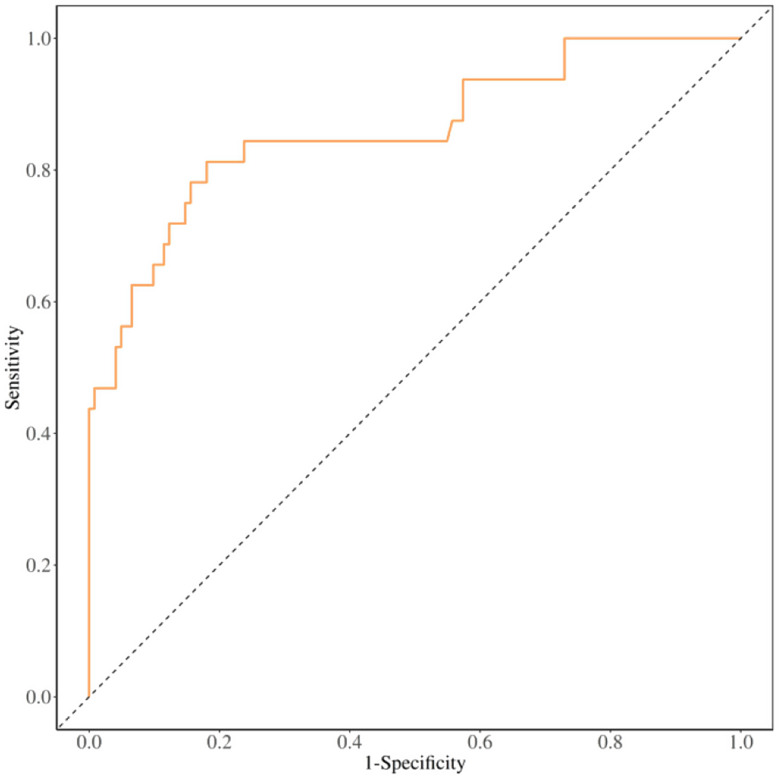
ROC curve

**Fig. 4:**
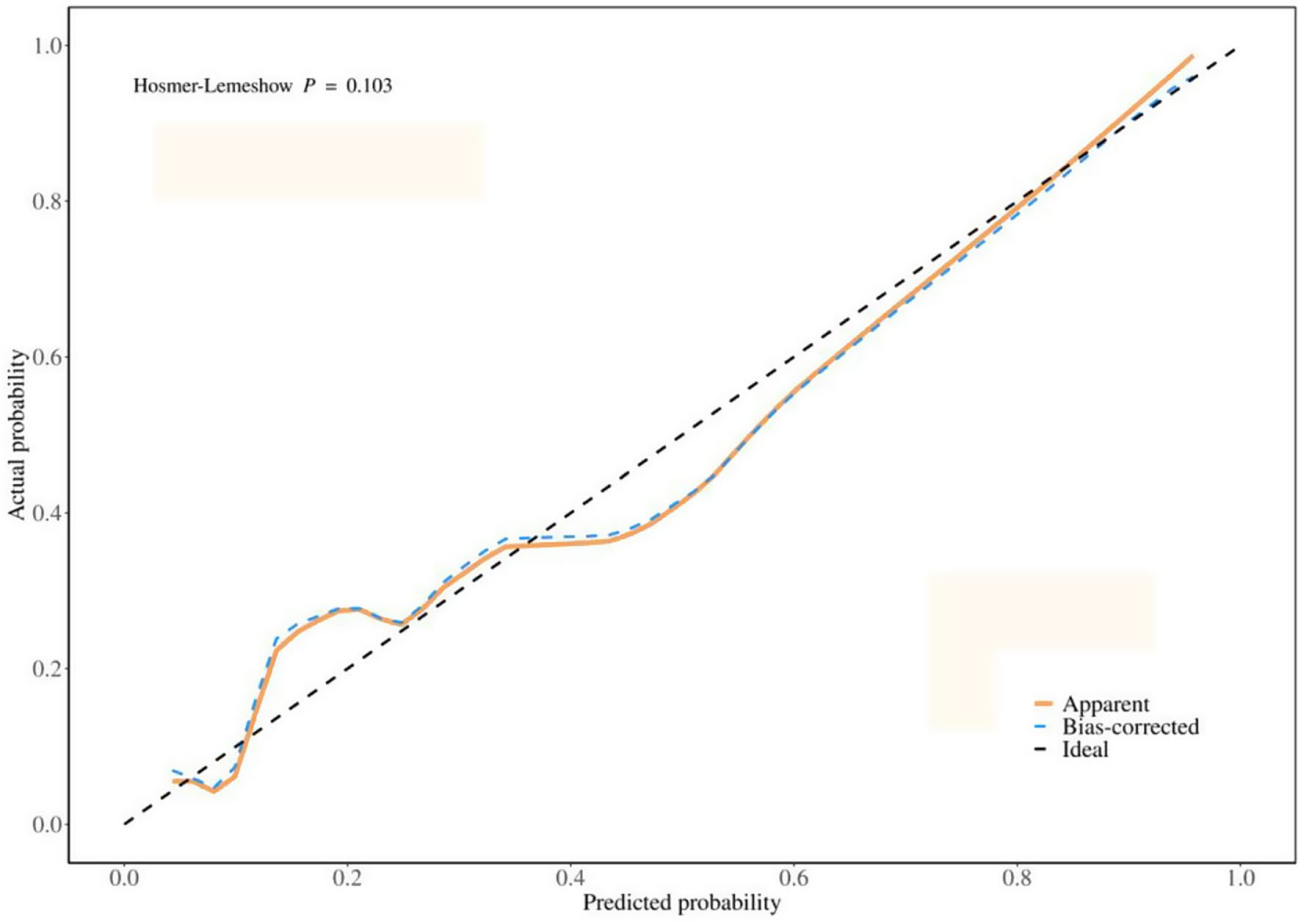
Calibration curve

Multivariate logistic regression analysis indicated that the absolute PCT value on postoperative day 2, WBC value on postoperative day 5, PCT variability from T4 to T1, and the number of days of mechanical ventilation significantly predicted POP, as demonstrated in Table 5. The results of the ROC analysis indicated that the model’s AUC was 0.860 (95% CI:0.778–0.942), with an accuracy of 0.818 (95% CI:0.748–0.876). The sensitivity was 0.820 (95%CI:0.751–0.888), and the specificity was 0.812 (95% CI:0.677–0.948). as demonstrated in Table 6(Fig. 3). Regarding the calibration curve results, the IDEAL curve exhibited a 45°upward trend, suggesting that the predicted probabilities of the model aligned well with the actual probabilities. Additionally, the Hosmer-Lemeshow test was employed to evaluate calibration, yielding a test value of 0.103 (*P* > 0.05), which indicates that the model was well-calibrated and capable of accurately assessing risk. (Fig. 4)

**Table 5:** Regression model identifying factors associated with lung infection within 7 days after CPB cardiac surgery

variant	β	SE	Wald χ^2^	*P*-value	OR (95% CI)
PCT T_2_	0.086	0.026	11.274	0.001	1.089 (1.036,1.145)
WBC T_5_	0.119	0.056	4.574	0.032	1.126 (1.010, 1.256)
PCT T_4 ~ 1_	0.427	0.164	6.764	0.009	1.533 (1.111, 2.114)
mechanical ventilation	0.235	0.051	21.212	< 0.001	1.265 (1.144,1.398)
constant	-3.697	0.673			

**Table 6:** AUC, accuracy, specificity, and sensitivity of the model

AUC (95%CI)	Accuracy (95%CI)	Sensitivity (95%CI)	Specificity (95%CI)	PPV (95%CI)	NPV (95%CI)	cut off
0.860 (0.778–0.942)	0.818 (0.748–0.876)	0.820(0.751–0.888)	0.812(0.677–0.948)	0.943(0.899–0.987)	0.542(0.401–0.683)	0.231

### Impact of POP on clinical outcomes

**Fig. 5:**
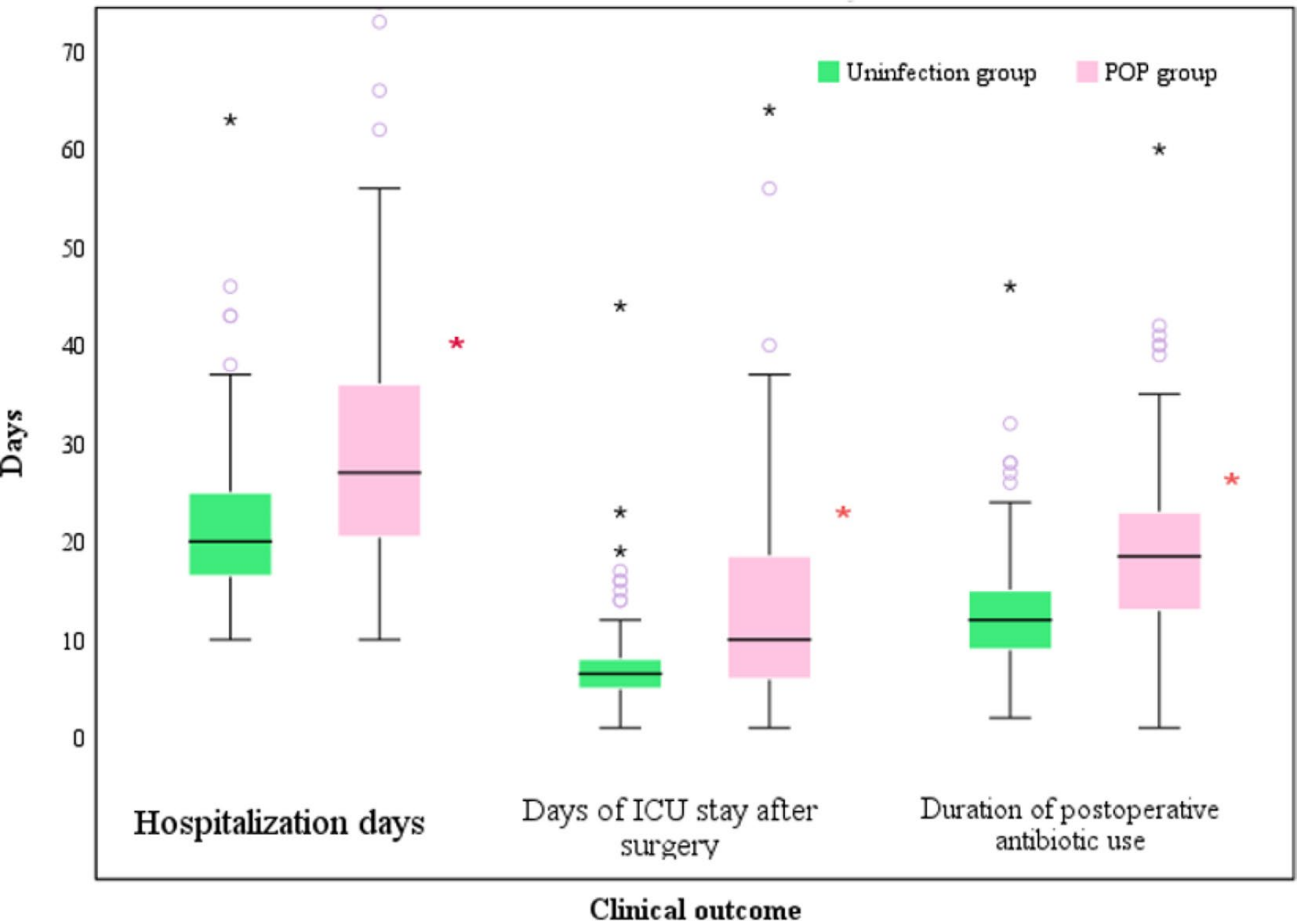
Comparison of different clinical outcomes between the two groups (red asterisks represent statistical differences between groups (*P* < 0.001))

Comparison of clinical outcomes between the two patient groups using box plots revealed that the total number of hospital days, length of postoperative ICU stay, and length of postoperative antimicrobial drug use were all significantly greater in the POP group compared to the uninfected group (*P* < 0.001) (Fig. 5).

## Discussion

The incidence of POP is influenced by various factors, including the type of surgery, the patient’s baseline health status, and nosocomial infection control measures [14, 15]. Given the absence of a uniform diagnostic standard, reported incidences of POP significantly vary across hospitals, with overall incidences ranging from 0.9 to 1.6%. However, certain studies indicate incidences as high as 15.8%, with postoperative bacterial pneumonia incidence at 21.6%, trailing only behind postoperative incision and urinary tract infections. In China, postoperative bacterial pneumonia was 21.6%, second only to postoperative incisional infections and urinary tract infections. The incidence of surgical POP significantly varies by site; however, major thoracic and upper abdominal surgeries tend to impair respiratory muscles, leading to a notably higher incidence of POP compared to other sites [16]. Regarding cardiac surgery, the incidence of POP also demonstrates variability, with previous literature reporting a range from as low as 2.1% to as high as 24.2% [17, 18]. The incidence of POP after cardiac surgery at our institution remained around 5% for three consecutive years, exhibiting a significant downward trend in 2023 and presenting as comparatively low against other relevant reports. This outcome can be attributed to the medical team’s efficient collaboration, stringent medical procedures, and ongoing quality improvement initiatives.

Although the incidence of pneumonia post-cardiac surgery remains relatively low, it’s crucial to recognize that, despite its infrequency, the severe complications that can ensue from pneumonia significantly impact patient recovery and long-term prognosis. Figure 5 illustrates that patients with POP experienced substantially longer hospital stays, intensive care unit (ICU) stays, and antimicrobial usage than the non-infected group. To identify lung infections early and implement timely interventions, this study evaluated the postoperative dynamics of infectious markers like PCT and their clinical diagnostic utility for POP. As shown in Table 2, key factors potentially affecting the study outcomes, including smoking history, surgery type, and comorbidities, were excluded. Preoperative nutritional status is a significant, influential factor [19]. This study incorporated the CONUT score, a less frequently addressed indicator in previous research on risk factors for postoperative cardiac infections. Previous studies primarily utilized nutritional indices like BMI, ALB, TLC, etc. Compared to a single laboratory marker, a composite marker offers a more comprehensive assessment of patients’ nutritional status [9]. However, this study found no significant difference in preoperative dietary status between the two groups.

PCT, a peptide precursor synthesized by thyroid C-cells, is increasingly utilized to assess the risk of systemic infection or sepsis. Recently, its application in medicine has expanded, particularly in predicting post-surgical infections, gaining considerable attention. PCT’s utility in predicting postoperative infections across various surgical disciplines—including organ transplantation [20], orthopedic [21], abdominal [22], neuro [23], and gynecological surgery [24]—has been well-documented. Interpreting calcitonin levels alongside other clinical indicators and assessing the patient’s overall condition aids in identifying high-risk patients. This study systematically assessed the patterns of calcitonin gene changes following extracorporeal cardiac surgery in adults to evaluate its diagnostic value in early pneumonia detection. Results indicated significantly higher calcitoninogen levels in patients with early POP, underscoring its potential as a diagnostic biomarker for early pneumonia. ROC curve analysis revealed that PCT exhibited the highest diagnostic accuracy among the studied parameters, making it an optimal indicator for the early diagnosis of lung infection post-cardiac surgery. Our study offers more detailed insights into calcitoninogen dynamics compared to existing research. In-depth analysis revealed that PCT levels peaked on the second postoperative day, with concentrations in the infected group significantly surpassing those in the control group at all measured time points. Additionally, WBC counts peaked on the second day, yet notable differences were observed on the fifth day among infected patients. Crucially, the rate of PCT variation was markedly higher in the infected group, further illustrating the significant impact of infection on PCT fluctuations. These observations offer vital insights for refining postoperative management and infection control protocols. Furthermore, our study incorporated leukocyte and PCT variability rates to prevent the over-interpretation of fluctuations in individual biomarkers. A study encompassing 423 patients who underwent cardiac surgery with CPB assessed the predictive value of combining CRP, WBC, and PCT levels for postoperative infections. It confirmed that the combined assessment of PCT and leukocyte levels during the initial three postoperative days accurately predicted infections up to 30 days following cardiac surgery, providing a robust tool for early detection and management of potential post-surgical infections [25].

In the multivariate analysis, the PCT level on day 2 post-surgery, WBC count on day 5 post-surgery, PCT variability on day 4, and the duration of mechanical ventilation were included. Notably, the duration of mechanical ventilation has been identified as an independent risk factor for POP in a range of surgical procedures, extending beyond cardiac surgery. This underscores the significance of monitoring mechanical ventilation duration alongside PCT levels and WBC counts as part of a comprehensive approach to assessing the risk of POP [7, 26]; this study demonstrated that the PCT concentration in patients within the infection group peaked in the second postoperative day, subsequently decreasing but remaining elevated above the standard threshold (> 0.5 ng/ml) on the fifth postoperative day. In contrast, the PCT concentration in the non-infection group continued to decline, falling within the normal range (< 0.5 ng/ml) by the fifth day. A significant difference in PCT concentrations was observed between infected and non-infected patients throughout the postoperative period. These findings align with previous research on adults [7]; however, studies involving pediatric patients have indicated no statistically significant difference in PCT concentration between infected and non-infected groups within the first three days post-surgery. Furthermore, PCT concentrations in both patient groups continue to decrease from the second day after surgery [27]. Considering the kinetics of PCT response to bacterial infections, possible explanations include: (1) a delayed response to infection, where PCT levels typically rise in response to systemic bacterial infections, making postoperative day 2 a more predictive marker for POP. This implies that a postoperative infection might require time to trigger a systemic response significant enough to manifest in PCT levels. This delay can be attributed to the time needed for the infection to establish itself and for the body to mount a systemic inflammatory response. (2) Specificity of PCT for bacterial infections: PCT shows heightened sensitivity to bacterial infections over other types, such as viral infections. Given that POP is predominantly bacterial, an elevated PCT level on postoperative day 2 is a specific marker for bacterial pneumonia. This specificity renders PCT an invaluable biomarker for the early detection of bacterial infections. (3) Surgical stress response: Surgery initiates an inflammatory response influencing the levels of various biomarkers, PCT included. However, the initial increase in inflammatory markers post-surgery may more likely reflect surgical stress than infection. By day 2, distinguishing between surgical and infection-induced inflammation may become more apparent, rendering PCT a more reliable infection marker [28]. ROC curve analysis results indicated that the PCT threshold on the second postoperative day was 3.45 ng/ml, suggesting clinicians may need to perform early diagnostic assessments. This threshold is notably higher than those reported in previous studies. For instance, a meta-analysis published in The Lancet Infectious Diseases identified a median cutoff value of PCT as a marker for diagnosing sepsis at 1.1 ng/mL [29]. Furthermore, the expert consensus on the clinical application of PCT in emergency care, published in China in 2012, offers detailed recommendations for PCT monitoring in common infectious diseases. It specifies that the threshold level for PCT to diagnose sepsis is > 0.5 ng/ml, the optimal threshold for diagnosing endocarditis is 2–3 ng/mL, and the median PCT level for bacterial infection is 1.84 ng/mL. A PCT cutoff value of 0.25 ng/mL is a critical indicator for initiating and discontinuing antibiotic therapy for lower respiratory tract infections [30]. A meta-analysis conducted in 2024, focusing on diagnosing adult cardiac postoperative infections, included 2,984 patients. The findings indicated that PCT is effective in ruling out uncertain infections. The analysis established an optimal threshold of 3 ng/ml, with the relevant time point as the second postoperative day (POD2) [31]. In conclusion, the significant predictive value of PCT levels on postoperative day 2 for POP underscores the importance of understanding the body’s temporal response to surgery and infection, enabling timely and appropriate clinical interventions. Furthermore, the leukocyte count on postoperative day 5 emerged as an independent risk factor for POP, a discovery potentially linked to our study’s inclusion criteria. Specifically, our study’s case group comprised only patients who developed pneumonia within the first 7 days following surgery. Within this group, leukocyte counts peaked by postoperative day 5, leading to generally higher leukocyte counts in the infected versus noninfected groups. This contrasts with prior studies where leukocytes were seldom considered an independent predictor of postoperative infection [32].

Another key finding of this study is that the diagnostic value of the absolute PCT level surpasses that of PCT variability and WBC count, diverging from the conclusions of prior research.PCT variability reflects the dynamic infection process and condition changes, with studies indicating that patients with postoperative infections typically exhibit higher PCT variability, marking a significant distinction from non-infected patients. Thus, PCT variability emerges as a crucial diagnostic marker for postoperative infection, potentially surpassing the absolute PCT level in diagnostic efficacy [7]. However, there remains no definitive conclusion regarding the superior diagnostic value of either metric. Additionally, this study reveals that calculating the PCT variation rate is complex, necessitating continuous PCT level monitoring and change rate calculation, thereby somewhat limiting its clinical utility. Conversely, the absolute PCT level provides rapidity, simplicity, and ease of operation, facilitating widespread clinical application. Regarding clinical applicability, we also plan to collaborate with the information department to integrate this early warning model with hospital information systems. Before clinical deployment, we must conduct rigorous multi-stage verification. We will initially examine various patient populations by utilizing a multicenter dataset encompassing diverse demographics and surgical protocols to assess the model’s robustness. This will be succeeded by prospective clinical testing in a realistic environment, where we will quantify sensitivity and specific indicators while monitoring workflow integration parameters, including real-life scenario indicators such as alarm fatigue and decision time. Additionally, it is crucial to conduct focus group interviews to evaluate clinician acceptance. Optimization based on a people-centered iterative system can enhance clinical practicality while preserving clinician autonomy.

However, this study has several limitations. Firstly, as a single-center retrospective study, the results might be influenced by specific surgical techniques and management strategies, thereby somewhat limiting their generalizability and necessitating further validation in broader clinical settings. Secondly, the analysis excluded some risk factors potentially associated with developing POP, such as disease severity. Thirdly, the study’s observed outcome was the incidence of pneumonia within 7 days postoperatively, whereas the typical diagnostic window for POP extends to 30 days post-surgery. This lack of follow-up to 30 days could introduce bias in classifying some cases as non-infected. Finally, due to the impact of medical insurance cost control policies, the dynamic monitoring of C-reactive protein (CRP) and interleukin-6 is not routinely conducted in clinical practice. In the future, we plan to consider the inclusion of cost-effective indicators such as the neutrophil/lymphocyte ratio (NLR), platelet/lymphocyte ratio (PLR) [33], and mid-regional pro-adrenomedullin [34] from a cost-effectiveness perspective. We will investigate the value of utilizing multiple indicators for combined diagnosis to enhance the sensitivity and specificity of POP diagnosis. Furthermore, the differing kinetics of these biomarkers can be leveraged to monitor treatment efficacy, thereby providing a more refined approach to managing postoperative infections.

## Conclusions

In conclusion, in the clinical management of patients undergoing cardiac surgery with CPB, the incidence of early POP is approximately 5%. The detection of PCT and WBC counts can effectively predict the occurrence of early POP, with the diagnostic value of PCT surpassing that of WBC and the variation rate of PCT. Specifically, the PCT level on postoperative day 2, WBC level on postoperative day 5, PCT variation rate on day 4, and mechanical ventilation duration were independent predictors of early POP. The study’s findings indicate that the optimal cut-off value for absolute PCT on postoperative day 2 is 3.45 ng/ml, which can serve as an alert for clinicians to conduct early diagnostic assessments.

## Electronic supplementary material

Below is the link to the electronic supplementary material.

Supplementary Material 1

Supplementary Material 2

## Data Availability

Data is provided within the supplementary information files. All data were collected by the Xinglin Real-Time Nosocomial Infection System and Intelligent Integrated Health(iih) platform.

## Abbreviations

PCT: Procalcitonin
CPB: Cardiopulmonary bypass
POP: Postoperative pneumonia
WBC: White blood cell
ROC: Receiver operating characteristic
CRP: C-reactive protein
OR: Odds ratio

## References

[1] Gerstein NS, Panikkath PV, Mirrakhimov AE, Lewis AE, Ram H. Cardiopulmonary bypass emergencies and intraoperative issues. J Cardiothorac Vasc Anesth. 2022;36(12):4505–22. 36100499 10.1053/j.jvca.2022.07.011

[2] Kallel S, Abid M, Jarraya A, Abdenadher M, Mnif E, Frikha I, et al. [Kinetics, diagnostic and prognostic value of procalcitonin after cardiac surgery]. Ann Biol Clin (Paris). 2012;70(5):567–80. 23047903 10.1684/abc.2012.0745

[3] Chen W, Zhong K, Guan Y, Zhang HT, Zhang H, Pan T, et al. Evaluation of the significance of interleukin-6 in the diagnosis of postoperative pneumonia: a prospective study. BMC Cardiovasc Disord. 2022;22(1):306. 35794529 10.1186/s12872-022-02744-0 PMC9261039

[4] Aïssou L, Sorbets E, Lallmahomed E, Goudot F-X, Pop N, Es-Sebbani S, et al. Prognostic and diagnostic value of elevated serum concentration of procalcitonin in patients with suspected heart failure. A review and meta-analysis. Biomarkers. 2018;23(5):407–13. 29465002 10.1080/1354750X.2018.1443511

[5] Sager R, Kutz A, Mueller B, Schuetz P. Procalcitonin-guided diagnosis and antibiotic stewardship revisited. BMC Med. 2017;15(1):15. 28114931 10.1186/s12916-017-0795-7 PMC5259962

[6] Abu Elyazed MM, El Sayed Zaki M. Value of procalcitonin as a biomarker for postoperative hospital-acquired pneumonia after abdominal surgery. Korean J Anesthesiol. 2017;70(2):177. 28367288 10.4097/kjae.2017.70.2.177 PMC5370307

[7] Jin H, Gu S-P, Wang Y, Pan K, Chen Z, Cao H-L, et al. Diagnosis value of procalcitonin variation on early pneumonia after adult cardiac surgery. Heart Surg Forum. 2021;24(4):E734–40. 34473021 10.1532/hsf.3987

[8] Bramante CT, Palzer EF, Rudser KD, Ryder JR, Fox CK, Bomberg EM, et al. BMI metrics and their association with adiposity, cardiometabolic risk factors, and biomarkers in children and adolescents. Int J Obes. 2022;46(2):359–65. 10.1038/s41366-021-01006-x PMC8926007 34718333

[9] Mureșan AV, Hălmaciu I, Arbănași EM, Kaller R, Arbănași EM, Budișcă OA, et al. Prognostic nutritional index, controlling nutritional status (CONUT) score, and inflammatory biomarkers as predictors of deep vein thrombosis, acute pulmonary embolism, and mortality in COVID-19 patients. Diagnostics. 2022;12(11):2757. 36428817 10.3390/diagnostics12112757 PMC9689150

[10] Hara M, Fujii T, Masuhara H, Kawasaki M, Tokuhiro K, Watanabe Y. The prognostic impact of the controlling nutritional status (CONUT) score in patients undergoing cardiovascular surgery. Gen Thorac Cardiovasc Surg. 2020;68(10):1142–7. 32248407 10.1007/s11748-020-01346-x

[11] Iosifidis E, Pitsava G, Roilides E. Ventilator-Associated pneumonia in neonates and children: A systematic analysis of diagnostic methods and prevention. Future Microbiol. 2018;13:1431–46. 30256161 10.2217/fmb-2018-0108

[12] Abbott TEF, Fowler AJ, Pelosi P, Gama de Abreu M, Møller AM, Canet J, et al. A systematic review and consensus definitions for standardised end-points in perioperative medicine: pulmonary complications. Br J Anaesth. 2018;120(5):1066–79. 29661384 10.1016/j.bja.2018.02.007

[13] Gambardella I, Gaudino MFL, Antoniou GA, Rahouma M, Worku B, Tranbaugh RF, et al. Single- versus multidose cardioplegia in adult cardiac surgery patients: A meta-analysis. J Thorac Cardiovasc Surg. 2020;160(5):1195–e120212. 31590948 10.1016/j.jtcvs.2019.07.109

[14] Bardia A, Blitz D, Dai F, Hersey D, Jinadasa S, Tickoo M, et al. Preoperative chlorhexidine mouthwash to reduce pneumonia after cardiac surgery: A systematic review and meta-analysis. J Thorac Cardiovasc Surg. 2019;158(4):1094–100. 30826096 10.1016/j.jtcvs.2019.01.014

[15] Nam K, Park J-B, Park WB, Kim NJ, Cho Y, Jang HS, et al. Effect of perioperative subglottic secretion drainage on Ventilator-Associated pneumonia after cardiac surgery: A retrospective, Before-and-After study. J Cardiothorac Vasc Anesth. 2021;35(8):2377–84. 33127285 10.1053/j.jvca.2020.09.126

[16] Chen B, Chen Y, Li C, Huang X, Zhou P, Wu A. Incidence and risk factors of postoperative pneumonia in abdominal operations patients at a teaching hospital in China. Infect Control Hosp Epidemiol. 2018;39(4):504–6. 29362004 10.1017/ice.2017.272

[17] Vera Urquiza R, Bucio Reta ER, Berríos Bárcenas EA, Choreño Machain T. Risk factors for the development of postoperative pneumonia after cardiac surgery. Arch Cardiol Mex. 2016;86(3):203–7. 26949195 10.1016/j.acmx.2015.12.005

[18] Allou N, Bronchard R, Guglielminotti J, Dilly MP, Provenchere S, Lucet JC, et al. Risk factors for postoperative pneumonia after cardiac surgery and development of a preoperative risk score**. Crit Care Med. 2014;42(5):1150–6. 24351376 10.1097/CCM.0000000000000143

[19] Tian Y, Zhu Y, Zhang K, Tian M, Qin S, Li X. Relationship between preoperative hypoalbuminemia and postoperative pneumonia following geriatric hip fracture surgery: A Propensity-Score matched and conditional logistic regression analysis. Clin Interv Aging. 2022;17:495–503. 35444412 10.2147/CIA.S352736 PMC9013674

[20] Nadziakiewicz P, Grochla M, Krauchuk A, Pióro A, Szyguła-Jurkiewicz B, Baca A, et al. Procalcitonin kinetics after heart transplantation and as a marker of infection in early postoperative course. Transpl Proc. 2020;52(7):2087–90. 10.1016/j.transproceed.2020.02.117 32305202

[21] Zhu X, Li K, Zheng J, Xia G, Jiang F, Liu H, et al. Usage of procalcitonin and sCD14-ST as diagnostic markers for postoperative spinal infection. J Orthop Traumatol. 2022;23(1):25. 35648304 10.1186/s10195-022-00644-9 PMC9160164

[22] Jerome E, McPhail M, Menon K. Diagnostic accuracy of procalcitonin and interleukin-6 for postoperative infection in major Gastrointestinal surgery: a systematic review and meta-analysis. Annals Royal Coll Surg Engl. 2022;104(8):561–70. 10.1308/rcsann.2022.0053 PMC9433179 36044921

[23] Yu Y, Li HJ. Diagnostic and prognostic value of procalcitonin for early intracranial infection after craniotomy. Braz J Med Biol Res. 2017;50(5):e6021. 28443989 10.1590/1414-431X20176021 PMC5441286

[24] Kong X, Liu K. The predictive value of PCT and other infection indicators in postoperative infection of epithelial ovarian cancer. Infect Drug Resist. 2023;16:1521–36. 36960392 10.2147/IDR.S399666 PMC10029970

[25] Heredia-Rodríguez M, Bustamante-Munguira J, Lorenzo M, Gómez-Sánchez E, Álvarez FJ, Fierro I, et al. Procalcitonin and white blood cells, combined predictors of infection in cardiac surgery patients. J Surg Res. 2017;212:187–94. 28550906 10.1016/j.jss.2017.01.021

[26] Fernández-Ugidos P, Barge‐Caballero E, Gómez‐López R, Paniagua‐Martin MJ, Barge‐Caballero G, Couto‐Mallón D, et al. In‐hospital postoperative infection after heart transplantation: risk factors and development of a novel predictive score. Transpl Infect Disease. 2019;21(4):e13104. 31077542 10.1111/tid.13104

[27] Li X, Wang X, Li S, Yan J, Li D. Diagnostic value of procalcitonin on early postoperative infection after pediatric cardiac surgery. Pediatr Crit Care Med. 2017;18(5):420–8. 28266954 10.1097/PCC.0000000000001118

[28] Arkader R. Procalcitonin does discriminate between sepsis and systemic inflammatory response syndrome. Arch Dis Child. 2005;91(2):117–20. 16326799 10.1136/adc.2005.077446 PMC2082702

[29] Wacker C, Prkno A, Brunkhorst FM, Schlattmann P. Procalcitonin as a diagnostic marker for sepsis: a systematic review and meta-analysis. Lancet Infect Dis. 2013;13(5):426–35. 23375419 10.1016/S1473-3099(12)70323-7

[30] Lee C-C, Kwa ALH, Apisarnthanarak A, Feng J-Y, Gluck EH, Ito A, et al. Procalcitonin (PCT)-guided antibiotic stewardship in Asia-Pacific countries: adaptation based on an expert consensus meeting. Clin Chem Lab Med (CCLM). 2020;58(12):1983–91. 31926074 10.1515/cclm-2019-1122

[31] Nicolotti D, Grossi S, Palermo V, Pontone F, Maglietta G, Diodati F, et al. Procalcitonin for the diagnosis of postoperative bacterial infection after adult cardiac surgery: a systematic review and meta-analysis. Crit Care. 2024;28(1):44. 38326921 10.1186/s13054-024-04824-3 PMC10848477

[32] Sharma P, Patel K, Baria K, Lakhia K, Malhotra A, Shah K, et al. Procalcitonin level for prediction of postoperative infection in cardiac surgery. Asian Cardiovasc Thorac Ann. 2016;24(4):344–9. 27002098 10.1177/0218492316640953

[33] Damar Çakırca T, Torun A, Çakırca G, Portakal RD. Role of NLR, PLR, ELR and CLR in differentiating COVID-19 patients with and without pneumonia. Int J Clin Pract. 2021;75(11):e14781. 34482573 10.1111/ijcp.14781 PMC8646493

[34] Miron M, Blaj M, Ristescu AI, Iosep G, Avădanei A-N, Iosep D-G, et al. Hospital-Acquired pneumonia and Ventilator-Associated pneumonia: A literature review. Microorganisms. 2024;12(1):213. 38276198 10.3390/microorganisms12010213 PMC10820465

